# Optimisation of a Multi-Functional Piezoelectric Component for a Climbing Robot

**DOI:** 10.3390/ma16145076

**Published:** 2023-07-18

**Authors:** Zachary J. Wegert, Anthony P. Roberts, Tirthankar Bandyopadhyay, Vivien J. Challis

**Affiliations:** 1 School of Mathematical Sciences, Queensland University of Technology, 2 George Street, Brisbane, QLD 4000, Australia; 2 The Robotics and Autonomous Systems Group, CSIRO, 1 Technology Ct, Pullenvale, QLD 4069, Australia

**Keywords:** structural optimisation, piezoelectric sensor, climbing robot

## Abstract

Force sensors on climbing robots give important information to the robot control system, however, off-the-shelf sensors can be both heavy and bulky. We investigate the optimisation of a lightweight integrated force sensor made of piezoelectric material for the multi-limbed climbing robot MAGNETO. We focus on three design objectives for this piezoelectric component. The first is to develop a lightweight component with minimal compliance that can be embedded in the foot of the climbing robot. The second objective is to ensure that the component has sensing capability to replace the off-the-shelf force sensor. Finally, the component should be robust for a range of climbing configurations. To this end, we focus on a compliance minimisation problem with constrained voltage and volume fraction. We present structurally optimised designs that satisfy the three main design criteria and improve upon baseline results from a reference component. Our computational study demonstrates that the optimisation of embedded robotic components with piezoelectric sensing is worthy of future investigation.

## 1. Introduction

Topology optimisation of macroscopic components has been applied to a wide array of industries including mechanical, civil, aerospace and biomedical engineering [1,2]. Recently, topology optimisation has been successfully applied to the design of robot components with an emphasis on weight reduction [3,4,5]. Other robotic applications include grippers (e.g., [6]), soft robotics (e.g., [7]), or a combination of the two (e.g., [8,9]). The importance of topology optimisation in the field of robotics is partly due to the requirement for mobile robots to be as light as possible. In particular, for climbing robots, weight reduction is key to increase payload.

The ability to sense the robot’s state and surrounding environment is also key to the design and control of robots. The use of piezoelectric materials as sensors is common (e.g., [10,11,12,13]) and more recently force direction and location sensing has been achieved through the use of designed piezoelectric materials [14]. There is also a growing body of research relating to the topology optimisation of piezoelectric devices and components. The majority of this work relates to optimising piezoelectric actuators or energy harvesting devices [6,15,16,17,18,19,20,21,22,23,24,25,26]. The design of piezoelectric sensors has also been considered, particularly the design of layered piezoelectric sensors with a view to optimising their dynamic vibration response [20,23,27]. Zheng et al. [28] also considered the design of a two-dimensional cantilever force sensor.

To date, the research establishes the potential of topology optimisation to obtain improved designs for piezoelectric components, leading us to consider the optimal design of a force-sensing piezoelectric component for a robotic application. In particular, we focus on applying topology optimisation to a specific robot component that requires multi-functional sensing and structural capabilities. A novel aspect of our work is that we do not consider a layered design and instead formulate the optimisation problem in a three-dimensional design domain. A further innovation of our approach is that we consider an optimised architectured piezoelectric material [29] as a possible base material for the optimisation problem.

**Figure 1:**
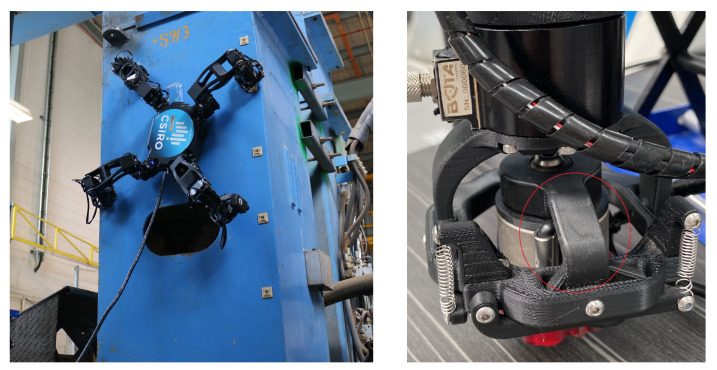
(**Left**): The multi-limbed climbing robot MAGNETO designed and built by CSIRO [30]. (**Right**): The robot’s foot with the component of interest circled in red. Images courtesy of Data61, CSIRO.

We consider the quasi-static design of a multi-functional sensing component for the multi-limbed climbing robot MAGNETO (Figure 1). MAGNETO, which was designed by CSIRO (Australia) [30], is a versatile robot designed for confined spaces such as complicated industrial structures. It uses four electromagnetised feet to climb structures as shown by the left image of Figure 1. In climbing applications the weight of a robot significantly affects the payload capacity. MAGNETO weighs 5.53 kg and is able to carry up to an additional 1.5 kg payload. To aid the robot, six-axis off-the-shelf force sensors are used in the limbs to measure the contact forces and detect whether the robot has a suitable grip on the wall. These sensors add significant weight to the design that restricts the payload capacity. As an alternative to these sensors, we consider the computational design of custom, lightweight, integrated force sensors made of piezoelectric material. The aim is to consider how such components could have both a structural role and act as sensors by sending electrical signals to the robot control system.

The component that we focus on optimising is located in the foot of MAGNETO as shown in the right image of Figure 1. We choose this component so that the voltage readings will reflect the forces acting on the larger structure. Furthermore, since there are eight of these components in total (two for each foot), the optimised piezoelectric components would give eight voltage readings that could be used to better inform MAGNETO’s control system. To benchmark our optimised designs we consider a reference component that resembles the existing component in the right of Figure 1.

We focus on a set of three design objectives for this piezoelectric component:

1. Mechanical properties: the component should be lightweight and have low compliance meaning that it will have a small deflection under loading.
2. Sensing capability: the component needs sensing capability to replace the off-the-shelf force sensor in each foot. As each force sensor weighs roughly 120 g, this should not only save weight but also provide information regarding the internal stress state of the foot.
3. Robustness to climbing configurations: the component should be functional for at least three actuation cases: horizontal movement, vertical movement, and inverted horizontal movement. These three cases correspond to the base test cases considered in the robot’s engineering design [30].

The remainder of this paper is organised as follows. In Section 2 we detail the problem description for our optimisation of an integrated piezoelectric force sensor for MAGNETO. In Section 3 we complete the necessary sensitivity analysis, while Section 4 discusses the numerical implementation. The results for both the reference component and optimised components are presented in Section 5. The discussion is presented in Section 6 and the concluding remarks in Section 7.

## 2. Methods

### 2.1. Design Domain

**Figure 2:**
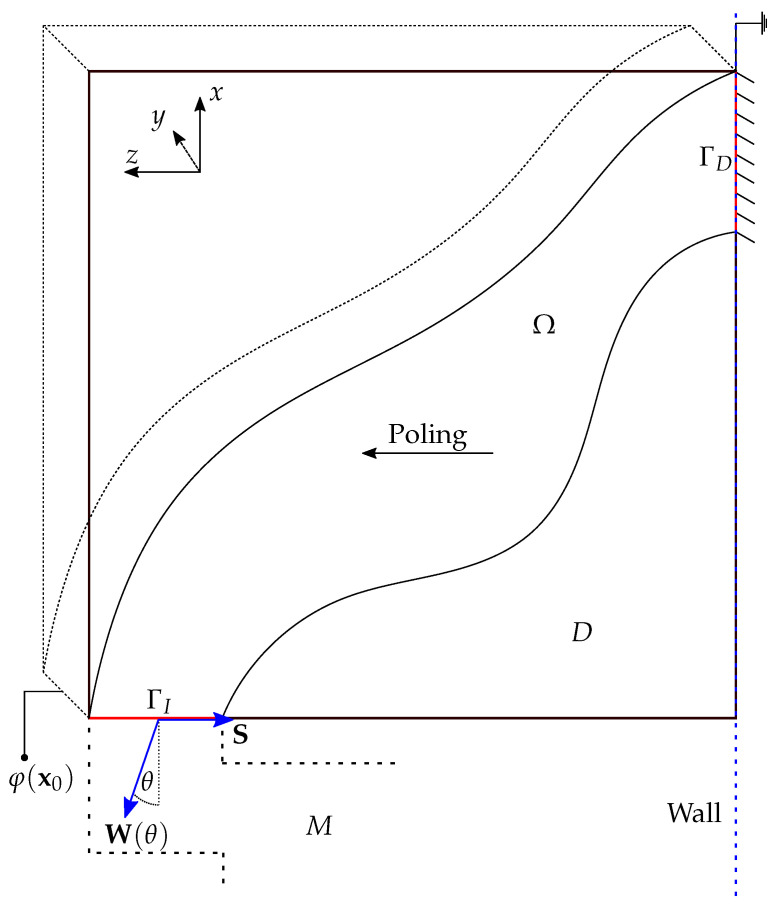
An illustration of the design domain *D* of the component where Ω is the material domain, $\Gamma_{D}$ is the zero Dirichlet potential and displacement boundary, $\Gamma_{I}$ is the Neumann stress boundary, and $\varphi (x_{0})$ is the voltage measured at the point $x_{0}$ positioned at the centre of $\Gamma_{I}$. It is important to note the indicated poling direction of the piezoelectric material.

In Figure 2 we present the design domain for the piezoelectric component with the following notation: *D* is the design domain, Ω is the material domain constructed from a piezoelectric material, $\Gamma_{D}$ is the grounded electrode and zero displacement boundary, $\Gamma_{I}$ is the boundary on which we apply the stress S for the contact force and W(θ) for the weight of the robot at an inclination θ. We measure the voltage φ at the centre point x${}_{0}$ of the boundary $\Gamma_{I}$. We denote the electromagnet at the centre of the foot by *M*. It is important to note the indicated poling direction of the piezoelectric material.

Neglecting body forces, body charge, and surface charge, we may write the governing equations of linear piezoelectricity as

(1a) $$\begin{array}{cc} -\sigma_{ij,i} & =0\;\mathrm{in}\;\Omega , \end{array}$$

(1b) $$\begin{array}{cc} D_{i,i} & =0\;\mathrm{in}\;\Omega , \end{array}$$

(1c) $$\begin{array}{cc} \sigma_{ij} & =C_{ijkl}^{E}\varepsilon_{kl}-e_{kij}E_{k}, \end{array}$$

(1d) $$\begin{array}{cc} D_{i} & =e_{ijk}\varepsilon_{jk}+\kappa_{ik}^{\varepsilon }E_{k}, \end{array}$$

(1e) $$\begin{array}{cc} \varepsilon_{ij} & =\frac{1}{2}\left(u_{i,j}+u_{j,i}\right), \end{array}$$

(1f) $$\begin{array}{cc} E_{i} & =-\varphi_{,i}, \end{array}$$

where $\sigma_{ij}$, $\varepsilon_{ij}$, $D_{i}$, $u_{i}$, $E_{i}$ and φ are the stress tensor, strain tensor, electric displacement vector, displacement vector, electric field vector, and electric potential, respectively, and $C_{ijkl}^{E}$, $\kappa_{ik}^{\varepsilon }$ and $e_{kij}$ are the elastic stiffness, dielectric, and piezoelectric coefficient tensors, respectively. The boundary conditions are given by

(2a) $$\begin{array}{cc} \; & u_{i}=0\;\mathrm{on}\;\Gamma_{D}, \end{array}$$

(2b) $$\begin{array}{cc} \; & \sigma_{ij}n_{i}=S_{j}+W_{j}\left(\theta \right)\;\mathrm{on}\;\Gamma_{I}, \end{array}$$

(2c) $$\begin{array}{cc} \; & \sigma_{ij}n_{i}=0\;\mathrm{on}\;\partial \Omega \setminus \left(\Gamma_{I}\cup \Gamma_{D}\right), \end{array}$$

(2d) $$\begin{array}{cc} \; & \varphi =0\;\mathrm{on}\;\Gamma_{D}, \end{array}$$

where $S_{j}$ is the surface traction due to magnet contact and $W_{j}\left(\theta \right)$ is the surface traction due to the weight of the robot under inclination θ.

The inclusion of the variable θ allows us to consider multiple loads in computationally designing the piezoelectric component. Three values of the angle θ correspond to configurations of the robot that are of particular interest:

- θ=−π/2: the robot is on a horizontal surface;
- θ=0: the robot is on a vertical surface; and
- θ=π/2: the robot is upside down on horizontal surface.

**Figure 3:**
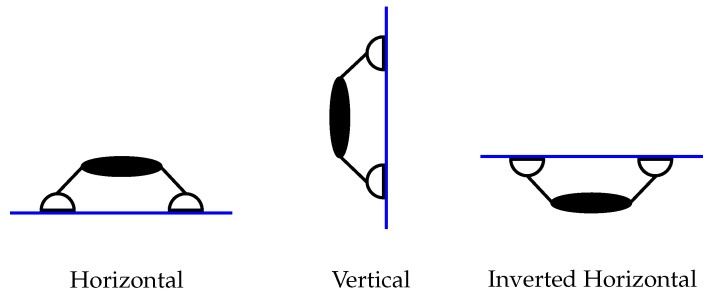
The climbing configurations considered in the optimisation problem where horizontal, vertical, and inverted horizontal correspond to θ=−π/2, θ=0, and θ=π/2, respectively.

Figure 3 shows an illustration of these configurations. For a robot of mass *m* under gravitational acceleration *g* we can write the vector W(θ)≡*W*${}_{j}\left(\theta \right)$ as

(3) $$W\left(\theta \right)=-\frac{mg}{8\mathrm{Area}(\Gamma_{I})}\left(\begin{array}{c} \cos\theta \\ 0\\ -\sin\theta \end{array}\right),$$

where the division by eight accounts for both the number of feet and the number of designed components on each foot.

### 2.2. Optimisation Problem

In this subsection we describe our optimisation problem and how it relates to the design objectives outlined in the Introduction (Section 1). We define two functionals of interest that will appear in our optimisation problem. For each functional we consider various inclinations $\theta =\theta_{\alpha }$ of the robot where α=1,⋯,m. While the mathematical development below is general, for the computational results we use the three loading angles ${\left\{\theta_{\alpha }\right\}}_{\alpha =1}^{3}=\left\{-\pi /2,0,\pi /2\right\}$ (as in Figure 3).

The first functional of interest is the classical compliance functional

(4) $$C_{\alpha }\left(u^{\left(\alpha \right)}\right)=\int_{\Gamma_{I}}\left(S+W\left(\theta_{\alpha }\right)\right)\cdot u^{\left(\alpha \right)}d\Gamma ,$$

where $u^{\left(\alpha \right)}$ is the displacement solution to the state equations for $\theta =\theta_{\alpha }$. The second functional of interest is the voltage φ measured at the point $x_{0}$ on $\Gamma_{I}$:

(5) $$J\left(\varphi^{\left(\alpha \right)}\right)=\int_{\Gamma_{I}}\varphi^{\left(\alpha \right)}\delta \left(x-x_{0}\right)d\Gamma ,$$

where δ(x) is the Dirac Delta function and $\varphi^{\left(\alpha \right)}$ is the electric potential solution to the state equations with $\theta =\theta_{\alpha }$.

We define our optimisation problem as minimisation of the weighted sum of compliance objectives for each inclination $\theta_{\alpha }$ subject to a constraint on the volume and weighted sum of voltages, and subject to the state equations. Mathematically this is written as:

(6) $$\begin{array}{cc} \underset{\rho_{e}}{\mathrm{minimise}}\;\; & \sum_{\alpha =1}^{m}a_{\alpha }C_{\alpha }\left(u^{\left(\alpha \right)}\right)\\ \mathrm{subject}\;\mathrm{to}\; & V\left(\rho_{e}\right)\leq V_{\max},\\ \; & \sum_{\alpha =1}^{m}b_{\alpha }J\left(\varphi^{\left(\alpha \right)}\right)\leq \varphi_{\min},\\ \; & h\left(\rho_{e}\right)=l_{\alpha }\left(\rho_{e}\right),\;\forall \alpha =1,\cdots ,m, \end{array}$$

where $\rho_{e}$ is the vector of element densities that are our design variables, $a_{\alpha }$ and $b_{\alpha }$ are weightings for the multi-load objectives and constraints, respectively, $V(\rho_{e})$ is the volume fraction, $V_{\max}$ is the maximum volume fraction, $\varphi_{\min}$ is the minimum voltage, and $h\left(\rho_{e}\right)=l_{\alpha }\left(\rho_{e}\right)$ represents the weak form for each loading angle. Care needs to be taken with the direction of the inequality for the voltage constraint because the sign of the voltage can change depending on the poling direction of the piezoelectric material. In our formulation the poling is in the positive *z* direction and the voltages measured at $x_{0}$ are negative. The weak form can be written as follows:

**Weak Form 1.**  For a loading angle $\theta_{\alpha }$, find $(u^{\left(\alpha \right)},\varphi^{\left(\alpha \right)})\in V\times Q$ such that

$$\begin{array}{cc} a_{uu}\left(C_{ijkl}^{E},u^{\left(\alpha \right)},v\right)-a_{u\varphi }\left(e_{ijk},\varphi^{\left(\alpha \right)},v\right) & =C_{\alpha }\left(v\right),\;\forall v\in V\\ a_{\varphi u}\left(e_{ijk},u^{\left(\alpha \right)},q\right)+a_{\varphi \varphi }\left(\kappa_{ik}^{\varepsilon },\varphi^{\left(\alpha \right)},q\right) & =0,\;\forall q\in Q \end{array}$$

where $Q=\{q\in H^{1}\left(\Omega \right):q=0\;\mathrm{on}\;\Gamma_{D}\}$, $V=\{v\in {\left[H^{1}\left(\Omega \right)\right]}^{3}:v=0\;\mathrm{on}\;\Gamma_{D}\}$, and

(7) $$\begin{array}{cc} \; & a_{uu}\left(C_{ijkl}^{E},u,v\right)=\int_{\Omega }C_{ijkl}^{E}\varepsilon_{kl}\left(u\right)\varepsilon_{ij}\left(v\right)d\Omega , \end{array}$$

(8) $$\begin{array}{cc} \; & a_{u\varphi }\left(e_{ijk},\varphi ,v\right)=\int_{\Omega }e_{kij}E_{k}\left(\varphi \right)\varepsilon_{ij}\left(v\right)d\Omega , \end{array}$$

(9) $$\begin{array}{cc} \; & a_{\varphi u}\left(e_{ijk},u,q\right)=\int_{\Omega }e_{ijk}\varepsilon_{jk}\left(u\right)E_{i}\left(q\right)d\Omega , \end{array}$$

(10) $$\begin{array}{cc} \; & a_{\varphi \varphi }\left(\kappa_{ik}^{\varepsilon },\varphi ,q\right)=\int_{\Omega }\kappa_{ik}^{\varepsilon }E_{k}\left(\varphi \right)E_{i}\left(q\right)d\Omega . \end{array}$$

We briefly discuss how the optimisation problem in Equation (6) reflects the design objectives for the piezoelectric component. The requirement of low compliance is reflected in the objective functional that is a linear combination of the compliance of the piezoelectric component for the different loading angles. A low weight is ensured via the constraint on the volume of material used. The sensing capability is included via the required minimum voltage under a linear combination of different load angles. Finally, inclusion of several loading angles in both the compliance and voltage functionals that appear in the optimisation problem reflects the need for the robot to be functional in several configurations.

## 3. Sensitivity Analysis

In this section we determine the sensitivity of the compliance and voltage functionals with respect to changes in the element densities $\rho_{e}$ that describe the material domain Ω. For this analysis we utilise the standard adjoint approach. The general outline of the adjoint approach for piezoelectricity has been presented by (Wegert [31], Chapter 3). We note that the sensitivity of the voltage functional is needed to impose the constraint on the weighted sum of voltages that appears in our optimisation problem, while the weighted sum of compliances appears in the optimisation objective. To simplify the notation in this section we consider a single inclination θ for both the compliance and voltage functionals. The necessary sensitivities for the optimisation objective and constraint that involve several inclinations is easily obtained via linearity.

### 3.1. Compliance

First we consider the single inclination compliance functional

$$\begin{array}{c} C\left(u\right)=\int_{\Gamma_{I}}f\cdot u(\rho_{e})d\Gamma , \end{array}$$

where f=S+W(θ). Suppose we let Λ∈V and $M_{1}\in Q$ be adjoint functions defined at a fixed $\rho_{e}$. Starting with C(u), we subtract the first equation in Weak Form 1 with $\Lambda_{1}$ in place of v and add the second equation in Weak Form 1 with $M_{1}$ in place of *q*:

(11) $$\begin{array}{cc} C(u) & =\int_{\Gamma_{I}}f\cdot ud\Gamma -a_{uu}\left(C_{ijkl}^{E},u,\Lambda_{1}\right)+a_{u\varphi }\left(e_{ijk},\varphi ,\Lambda_{1}\right)+C\left(\Lambda_{1}\right)\\ \; & \;+a_{\varphi u}\left(e_{ijk},u,M_{1}\right)+a_{\varphi \varphi }\left(\kappa_{ik}^{\varepsilon },\varphi ,M_{1}\right). \end{array}$$

Differentiating then gives

(12) $$\begin{array}{cc} C^{\prime }\left(u\right) & =\int_{\Gamma_{I}}f\cdot \frac{\partial u}{\partial \rho_{e}}d\Gamma -a_{uu}\left(C_{ijkl}^{E},\frac{\partial u}{\partial \rho_{e}},\Lambda_{1}\right)+a_{u\varphi }\left(e_{ijk},\frac{\partial \varphi }{\partial \rho_{e}},\Lambda_{1}\right)\\ \; & \;+a_{\varphi u}\left(e_{ijk},\frac{\partial u}{\partial \rho_{e}},M_{1}\right)+a_{\varphi \varphi }\left(\kappa_{ik}^{\varepsilon },\frac{\partial \varphi }{\partial \rho_{e}},M_{1}\right)\\ \; & \;-a_{uu}\left(\frac{\partial C_{ijkl}^{E}}{\partial \rho_{e}},u,\Lambda_{1}\right)+a_{u\varphi }\left(\frac{\partial e_{ijk}}{\partial \rho_{e}},\varphi ,\Lambda_{1}\right)\\ \; & \;+a_{\varphi u}\left(\frac{\partial e_{ijk}}{\partial \rho_{e}},u,M_{1}\right)+a_{\varphi \varphi }\left(\frac{\partial \kappa_{ik}^{\varepsilon }}{\partial \rho_{e}},\varphi ,M_{1}\right). \end{array}$$

Setting the first two lines to zero to remove terms that are difficult to compute gives weak form equations for $\Lambda_{1}$ and $M_{1}$:

**Weak Form 2.**  Find $(\Lambda_{1},M_{1})\in V\times Q$ such that

$$\begin{array}{cc} a_{uu}\left(C_{ijkl}^{E},\Lambda_{1},v\right)-a_{u\varphi }\left(e_{ijk},M_{1},v\right) & =C(v),\;\forall v\in V\\ a_{\varphi u}\left(e_{ijk},\Lambda_{1},q\right)+a_{\varphi \varphi }\left(\kappa_{ik}^{\varepsilon },M_{1},q\right) & =0,\;\forall q\in Q. \end{array}$$

This is exactly the weak form for our state equations (c.f., Weak Form 1). The problem is therefore self-adjoint with $\Lambda_{1}=u$ and $M_{1}=\varphi$, and the sensitivity of the compliance is given by

(13) $$\begin{array}{cc} C^{\prime }\left(u\right) & =-a_{uu}\left(\frac{\partial C_{ijkl}^{E}}{\partial \rho_{e}},u,u\right)+a_{\varphi u}\left(\frac{\partial e_{ijk}}{\partial \rho_{e}},u,\varphi \right)\\ \; & \;+a_{u\varphi }\left(\frac{\partial e_{ijk}}{\partial \rho_{e}},\varphi ,u\right)+a_{\varphi \varphi }\left(\frac{\partial \kappa_{ik}^{\varepsilon }}{\partial \rho_{e}},\varphi ,\varphi \right). \end{array}$$

In FE notation this is written as

(14) $$\begin{array}{c} \frac{\partial C}{\partial \rho_{e}}=-U^{e}\frac{\partial K_{uu}^{e}}{\partial \rho_{e}}U^{e}+U^{e}\frac{\partial K_{u\varphi }^{e}}{\partial \rho_{e}}\Phi^{e}+\Phi^{e}\frac{\partial K_{\varphi u}^{e}}{\partial \rho_{e}}U^{e}+\Phi^{e}\frac{\partial K_{\varphi \varphi }^{e}}{\partial \rho_{e}}\Phi^{e} \end{array}$$

where $K_{uu}^{e},K_{u\phi }^{e},K_{\phi u}^{e}$ and $K_{\phi \phi }^{e}$ are the element stiffness matrices for each bi-linear form and $U^{e}$ and $\Phi^{e}$ are the FE element solution vectors.

### 3.2. Voltage

Next we consider the single inclination voltage functional

(15) $$J\left(\varphi \right)=\int_{\Gamma_{I}}\varphi \delta (x-x_{0})d\Gamma ,$$

where δ(x) is the Dirac Delta function.

Suppose we let $\Lambda_{2}\in V$ and $M_{2}\in Q$ be adjoint functions defined at a fixed $\rho_{e}$. Similar to the above, starting with J(u) we add the first equation in Weak Form 1 with $\Lambda_{2}$ in place of v and subtract the second equation in Weak Form 1 with $M_{2}$ in place of *q*:

(16) $$\begin{array}{cc} J(\varphi ) & =\int_{\Gamma_{I}}\varphi \delta (x-x_{0})d\Gamma +a_{uu}\left(C_{ijkl}^{E},u,\Lambda_{2}\right)-a_{u\varphi }\left(e_{ijk},\varphi ,\Lambda_{2}\right)-C\left(\Lambda_{2}\right)\\ \; & \;-a_{\varphi u}\left(e_{ijk},u,M_{2}\right)-a_{\varphi \varphi }\left(\kappa_{ik}^{\varepsilon },\varphi ,M_{2}\right). \end{array}$$

Differentiating gives

(17) $$\begin{array}{cc} J^{\prime }\left(\varphi \right) & =\int_{\Gamma_{I}}\frac{\partial \varphi }{\partial \rho_{e}}\delta \left(x-x_{0}\right)d\Gamma +a_{uu}\left(C_{ijkl}^{E},\frac{\partial u}{\partial \rho_{e}},\Lambda_{2}\right)-a_{u\varphi }\left(e_{ijk},\frac{\partial \varphi }{\partial \rho_{e}},\Lambda_{2}\right)\\ \; & \;-a_{\varphi u}\left(e_{ijk},\frac{\partial u}{\partial \rho_{e}},M_{2}\right)-a_{\varphi \varphi }\left(\kappa_{ik}^{\varepsilon },\frac{\partial \varphi }{\partial \rho_{e}},M_{2}\right)\\ \; & \;+a_{uu}\left(\frac{\partial C_{ijkl}^{E}}{\partial \rho_{e}},u,\Lambda_{2}\right)-a_{u\varphi }\left(\frac{\partial e_{ijk}}{\partial \rho_{e}},\varphi ,\Lambda_{2}\right)\\ \; & \;-a_{\varphi u}\left(\frac{\partial e_{ijk}}{\partial \rho_{e}},u,M_{2}\right)-a_{\varphi \varphi }\left(\frac{\partial \kappa_{ik}^{\varepsilon }}{\partial \rho_{e}},\varphi ,M_{2}\right). \end{array}$$

Setting the first two lines to zero gives a weak form for the adjoint functions $\Lambda_{2}$ and $M_{2}$:

**Weak Form 3.**  Find $(\Lambda_{2},M_{2})\in V\times Q$ such that

$$\begin{array}{cc} a_{uu}\left(C_{ijkl}^{E},\Lambda_{2},v\right)-a_{u\varphi }\left(e_{ijk},M_{2},v\right) & =0,\;\forall v\in V\\ a_{\varphi u}\left(e_{ijk},\Lambda_{2},q\right)+a_{\varphi \varphi }\left(\kappa_{ik}^{\varepsilon },M_{2},q\right) & =J(q),\;\forall q\in Q. \end{array}$$

Solving this weak form for the adjoint functions $\Lambda_{2}$ and $M_{2}$ allows us to calculate the sensitivity of J(φ) as

(18) $$\begin{array}{cc} J^{\prime }\left(\varphi \right) & =a_{uu}\left(\frac{\partial C_{ijkl}^{E}}{\partial \rho_{e}},u,\Lambda_{2}\right)-a_{\varphi u}\left(\frac{\partial e_{ijk}}{\partial \rho_{e}},u,M_{2}\right)\\ \; & \;-a_{u\varphi }\left(\frac{\partial e_{ijk}}{\partial \rho_{e}},\varphi ,\Lambda_{2}\right)-a_{\varphi \varphi }\left(\frac{\partial \kappa_{ik}^{\varepsilon }}{\partial \rho_{e}},\varphi ,M_{2}\right). \end{array}$$

In FE notation this is written as

(19) $$\begin{array}{c} \frac{\partial J}{\partial \rho_{e}}=U^{e}\frac{\partial K_{uu}^{e}}{\partial \rho_{e}}\Lambda^{e}-U^{e}\frac{\partial K_{u\varphi }^{e}}{\partial \rho_{e}}M^{e}-\Phi^{e}\frac{\partial K_{\varphi u}^{e}}{\partial \rho_{e}}\Lambda^{e}-\Phi^{e}\frac{\partial K_{\varphi \varphi }^{e}}{\partial \rho_{e}}M^{e} \end{array}$$

where $K_{uu}^{e},K_{u\phi }^{e},K_{\phi u}^{e},K_{\phi \phi }^{e},U^{e}$ and $\Phi^{e}$ are as previously, and $\Lambda^{e}$ and $M^{e}$ are the element FE vectors associated with $\Lambda_{2}$ and $M_{2}$, respectively. It should be noted that this result matches [28] up to notation and agrees with finite difference calculations.

## 4. Numerical Implementation

### 4.1. Design Parameters

We take the size of the design domain *D* to be 25 mm, 10 mm, and 25 mm for the *x*, *y*, and *z* directions, respectively. This is approximately the size of the existing component on MAGNETO. We let the boundary $\Gamma_{I}$ have length 6.25 mm in the *z* direction and span the domain in the *y* direction. Likewise, the boundary $\Gamma_{D}$ is taken to have length 6.25 mm in the *x* direction and span the domain in the *y* direction.

For the Neumann boundary conditions on $\Gamma_{I}$ we take W(θ) as defined in Equation (3) with m=5.53 kg and g=9.81 m/s^2^. The contact stress S is chosen to be given by S=−2|W|(0,0,1) where |W| is the magnitude of W(θ), which is independent of θ. The coefficient of two on the contact stress is chosen so that the robot can withstand actuation failure where only two legs are connected to a horizontal wall.

We use three loading angles ${\left\{\theta_{\alpha }\right\}}_{\alpha =1}^{3}=\left\{-\pi /2,0,\pi /2\right\}$ as mentioned previously. The weighting $a_{\alpha }$ on the compliance objective is taken to be $a_{\alpha }=\left\{\frac{1}{3},\frac{1}{3},\frac{1}{3}\right\}$, meaning that our objective is the average compliance from the three configurations. The weighting on the sum of voltages is similarly chosen to be $b_{\alpha }=\left\{\frac{1}{3},\frac{1}{3},\frac{1}{3}\right\}$. We require that the average voltage should be less than $\varphi_{\min}=-137$ volts. This value is chosen based on the average voltage result for a reference component as discussed further below (Section 5.1). We consider two values for the maximum volume fraction and solve the optimisation problem for both of the cases $V_{\max}=0.5$ and $V_{\max}=0.35$.

### 4.2. Base Materials

We consider two different base materials for solving the optimisation problem. The first is *z*-poled PZT-5A, which has been used for previous piezoelectric optimisation work [29,32]. The material PZT-5A has a density of 7.8 g/cm^3^. The piezoelectric coefficients for this material are given in Voigt notation by (e.g., [32])

(20) $$\left[C_{pq}^{E,0}\right]=(\begin{array}{cccccc} 12.04 & 7.52 & 7.51 & 0.0 & 0.0 & 0.0\\ 7.52 & 12.04 & 7.51 & 0.0 & 0.0 & 0.0\\ 7.51 & 7.51 & 11.09 & 0.0 & 0.0 & 0.0\\ 0.0 & 0.0 & 0.0 & 2.1 & 0.0 & 0.0\\ 0.0 & 0.0 & 0.0 & 0.0 & 2.1 & 0.0\\ 0.0 & 0.0 & 0.0 & 0.0 & 0.0 & 2.3 \end{array})\times 10^{10}\;\left(N/m^{2}\right),$$

(21) $$\left[e_{ip}^{0}\right]=(\begin{array}{cccccc} 0.0 & 0.0 & 0.0 & 0.0 & 12.3 & 0.0\\ 0.0 & 0.0 & 0.0 & 12.3 & 0.0 & 0.0\\ -5.4 & -5.4 & 15.8 & 0.0 & 0.0 & 0.0 \end{array})\;\left(C/m^{2}\right),$$

(22) $$\left[\kappa_{ij}^{\varepsilon ,0}\right]=(\begin{array}{ccc} 4.78 & 0.0 & 0.0\\ 0.0 & 4.78 & 0.0\\ 0.0 & 0.0 & 7.35 \end{array})\times 10^{-9}\;\left(F/m\right).$$

We also consider a periodic piezoelectric material that was optimised for a linear combination of stiffness and piezoelectric properties in earlier work [29]. Specifically, we use the open-cell optimised material with the highest bulk modulus and a volume fraction of 50% presented in Wegert et al. [29].

**Figure 4:**
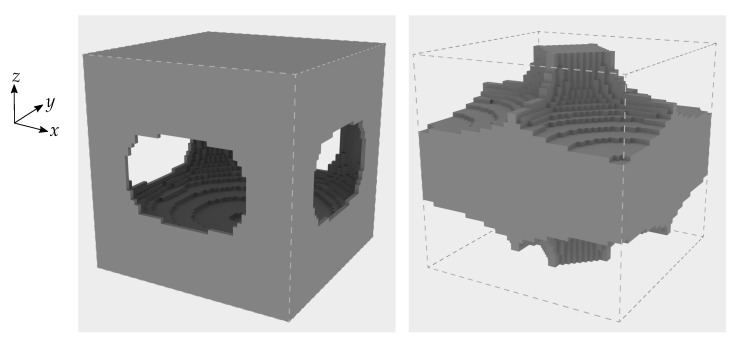
Open-cell optimised material with a volume fraction of 50%. The right image shows the same material where the centre of the base cell has been shifted by half the base cell edge length along each coordinate direction, enabling another view of the same microstructure.

Figure 4 shows the layout of this optimised base material. We choose this particular base cell for its potential manufacturability; closed-cell materials cannot be created via additive manufacturing. We ascribe the material properties of PZT-5A to the solid phase of this optimised base cell. The density of the optimised material is therefore 3.9 g/cm${}^{3}$ and its effective material constants are given by

(23) $$\left[C_{pq}^{E,0}\right]=(\begin{array}{cccccc} 4.365 & 2.284 & 0.4357 & 0.0 & 0.0 & 0.0\\ 2.284 & 4.365 & 0.4357 & 0.0 & 0.0 & 0.0\\ 0.4357 & 0.4357 & 0.7867 & 0.0 & 0.0 & 0.0\\ 0.0 & 0.0 & 0.0 & 0.1721 & 0.0 & 0.0\\ 0.0 & 0.0 & 0.0 & 0.0 & 0.1721 & 0.0\\ 0.0 & 0.0 & 0.0 & 0.0 & 0.0 & 1.003 \end{array})\times 10^{10}\;\left(N/m^{2}\right),$$

(24) $$\left[e_{ip}^{0}\right]=(\begin{array}{cccccc} 0.0 & 0.0 & 0.0 & 0.0 & 0.8596 & 0.0\\ 0.0 & 0.0 & 0.0 & 0.8596 & 0.0 & 0.0\\ -0.07159 & -0.07159 & 2.585 & 0.0 & 0.0 & 0.0 \end{array})\;\left(C/m^{2}\right),$$

(25) $$\left[\kappa_{ij}^{\varepsilon ,0}\right]=(\begin{array}{ccc} 5.059 & 0.0 & 0.0\\ 0.0 & 5.059 & 0.0\\ 0.0 & 0.0 & 1.077 \end{array})\times 10^{-9}\;\left(F/m\right).$$

Considering both the solid and optimised PZT-5A base materials for the optimisation problem allows us to computationally explore the potential benefits of using architectured open-cell piezoelectric materials.

### 4.3. Discretisation and Finite Element Method

We discretise the design domain into 40×10×40 linear hexahedral finite elements that are 0.625 mm ×1.0 mm ×0.625 mm in size and are each assigned a density $\rho_{e}$. This number of elements provides significant design freedom while keeping computational costs at a manageable level. In particular, design freedom is needed in the *x* and *z* directions, and less so in the *y* direction. We note that the number of finite element calculations is significant; at each iteration of the optimisation algorithm two finite element solutions are required for each inclination $\theta_{\alpha }$. One of these solves Weak Form 1, while the other solves Weak Form 3 that arises in solving for the adjoint functions that are needed to compute the sensitivity of the measured voltage. The initial element density $\rho_{e}$ for each element is chosen to be uniform at the required volume fraction.

For the finite element and sensitivity calculations we use the finite element package Gridap that is written in the programming language Julia [33]. The power of Gridap comes from its generality and syntax: it is able to solve a wide range of PDEs using syntax that corresponds very closely with the mathematical notation. We solve the resulting linear systems using an incomplete LU preconditioned conjugate gradient method with a drop tolerance of 4 for the preconditioner and a relative tolerance of $10^{-13}$ for the solver. It is important to stress that although the minimal residual method (MINRES) preconditioned with an incomplete Cholesky decomposition should theoretically be more efficient, we have found that conjugate gradient with an incomplete LU preconditioner is more efficient for a GPU implementation.

### 4.4. Topology Optimisation Algorithm

The topology optimisation algorithm used for the optimisation of the piezoelectric component is similar to that described previously by the authors for the design of periodic piezoelectric materials [29]. In the below we outline key details of the approach, some of which differ from our earlier work due to the piezoelectric component optimisation problem we are solving in the current paper.

Topology optimisation is achieved via the Solid Isotropic Material with Penalisation (SIMP) method [1], where the design variables are the density $\rho_{e}\in \left[0,1\right]$ of each element within the design domain. The SIMP material law for the piezoelectric material tensors within each element are given by

(26) $$\begin{array}{cc} C_{ijkl}^{E}\left(\rho_{e}\right) & =\rho_{e}^{p_{C}}C_{ijkl}^{E,0} \end{array}$$

(27) $$\begin{array}{cc} e_{ijk}\left(\rho_{e}\right) & =\rho_{e}^{p_{e}}e_{ijk}^{0} \end{array}$$

(28) $$\begin{array}{cc} \kappa_{ij}\left(\rho_{e}\right) & =\rho_{e}^{p_{\kappa }}\kappa_{ij}^{\varepsilon ,0}. \end{array}$$

Here $C_{ijkl}^{E,0}$, $e_{ijk}^{0}$ and $\kappa_{ij}^{\varepsilon ,0}$ are the piezoelectric tensors of the appropriate base material (either solid PZT-5A or optimised PZT-5A as described above), and $p_{C}$, $p_{e}$ and $p_{\kappa }$ are the penalisation exponents assosicated with each material property tensor. As described in our earlier work [29], the penalisation exponents for the SIMP method with piezoelectric materials must be chosen carefully. We use the appropriate values

(29) $$p_{C}=4,\;p_{e}=5\;\mathrm{and}\;p_{\kappa }=5.$$

Even with these appropriate penalisation exponents, optimised designs contain intermediate densities. This issue is addressed via a post-processing optimisation problem, where after 300 iterations of the optimisation algorithm the penalty term

(30) $$W\left(\rho_{e}\right)=\int_{\Omega }4\rho_{e}\left(1-\rho_{e}\right)d\Omega$$

is added to the optimisation objective. If the change between subsequent iterations is less than 0.5% and intermediate densities are still present, the post-processing optimisation problem is restarted with a larger coefficient of this penalty term until no intermediate densities are present. This process is as described previously [29] and effectively removes intermediate density elements.

Filtering of sensitivities using the standard mesh-independence filter [1] is employed with a filter radius of 1.5 elements. Such a filter is needed to prevent checkerboarding due to our compliance optimisation objective. We use the method of moving asymptotes (MMA) optimiser [34,35] where the magnitude of the objective and constraints are scaled during the optimisation process so that they are of similar size. The element density design variables have minimum value $10^{-7}$, maximum value 1.0, and the following MMA parameters are used move: 0.1, asyinit: 0.1 and asyincr: 1.0. These are relatively conservative choices that prevent the optimiser from too aggressively adding or removing material  [29,35].

## 5. Results

In the following we give computational results for a reference component and subsequently present our optimisation results.

### 5.1. Reference Component

**Figure 5:**
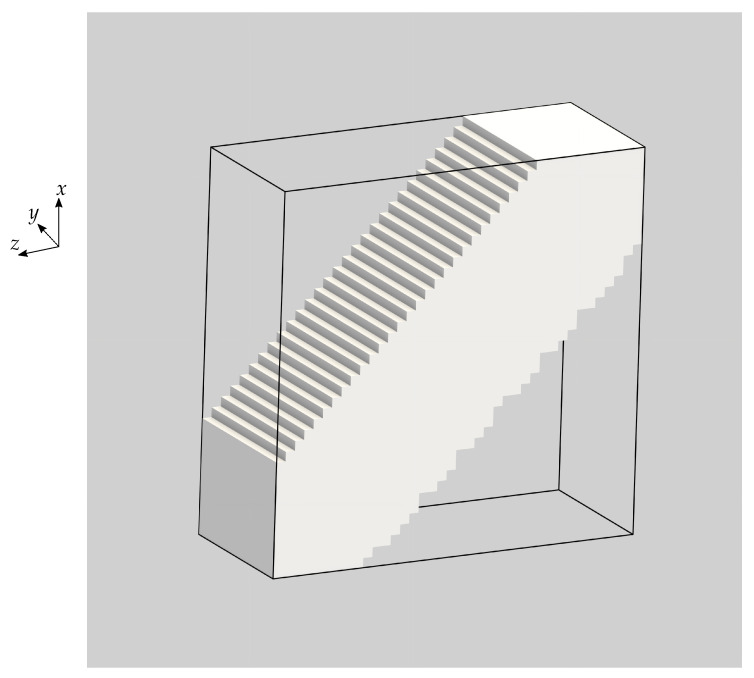
A visualisation of the reference component that is compared to the optimisation results. The volume fraction of the reference component is 0.4681.

We briefly present and discuss the computational results for a reference component made of the two base materials described above (Section 4.2) along with the standard three-dimensional printing material acrylonitrile butadiene styrene (ABS). These results will be useful for comparison with our optimisation results. The geometry of this reference component is chosen to approximate the existing component (right image of Figure 1) and its discretised domain is visualised in Figure 5.

ABS has a density of 1.1 g/cm${}^{3}$. The elastic stiffness tensor for the ABS material in Voigt notation is given by

(31) $$\left[C_{pq}\right]=(\begin{array}{cccccc} 0.4245 & 0.2493 & 0.2493 & 0.0 & 0.0 & 0.0\\ 0.2493 & 0.4245 & 0.2493 & 0.0 & 0.0 & 0.0\\ 0.2493 & 0.2493 & 0.4245 & 0.0 & 0.0 & 0.0\\ 0.0 & 0.0 & 0.0 & 0.08759 & 0.0 & 0.0\\ 0.0 & 0.0 & 0.0 & 0.0 & 0.08759 & 0.0\\ 0.0 & 0.0 & 0.0 & 0.0 & 0.0 & 0.08759 \end{array})\times 10^{10}\;\left(N/m^{2}\right),$$

which is generated via a Young’s modulus and Poisson’s ratio of E=2.4 GPa and ν=0.37, respectively [36]. ABS does not have piezoelectric properties. The purpose of this computation is to enable comparison of the compliance and weight of the reference and optimised piezoelectric components with the approximate compliance and weight of the existing part.

**Figure 6:**
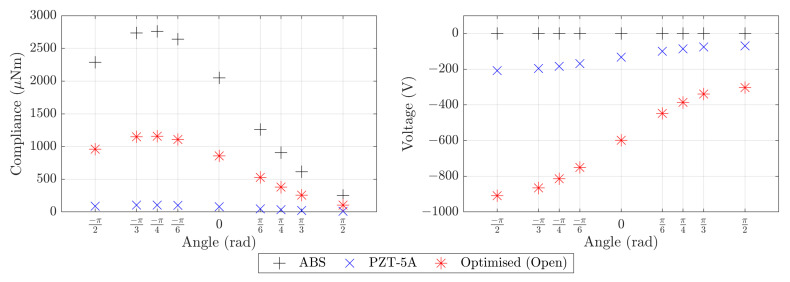
Compliance and voltage computational results for the reference component. The results are given for three base materials: ABS; PZT-5A; and the open-cell optimised material made of PZT-5A. The compliance and voltage are shown for a number of angles in addition to the angles of −π/2, 0, and π/2 that will be considered in our optimisation problem.

Table 1 details the mass, compliance and voltage for the reference component for each base material and inclinations of −π/2,0 and π/2. The data in Table 1 indicate that both the compliance and voltage of the reference component are much smaller when the robot is upside down. This is due to the fact that the stresses acting on $\Gamma_{I}$ are in opposite directions and somewhat cancel in this configuration (see Figure 2). Figure 6 shows the reference component compliance and voltage for each base material and a range of inclination angles between −π/2 and π/2.

### 5.2. Optimised Components

In this section we present our computational results for topology optimised components with PZT-5A and the open-cell optimised PZT-5A as base materials. As mentioned previously, we consider three configurations via the inclination angles −π/2, 0, and π/2 included in the optimisation problem and we consider two maximum volume fractions for each base material, $V_{\max}=0.5$ and $V_{\max}=0.35$.

**Figure 7:**
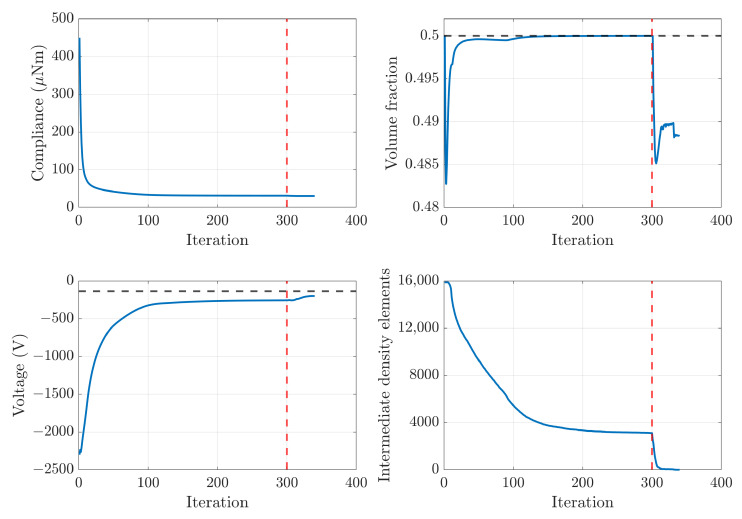
Optimisation history for the PZT-5A component with maximum volume fraction $V_{\max}=0.5$. The upper left subplot shows average compliance, the upper right shows volume fraction, the lower left shows average voltage and the lower right shows the number of intermediate density elements. The horizontal black dotted lines show the threshold for the volume fraction and voltage constraint in the upper right and lower left subplots, respectively. The red dotted line in each subplot indicates the iteration at which the post-processing starts and the optimisation objective subsequently explicitly penalises intermediate densities.

In Figure 7 we present the optimisation history for optimisation of the piezoelectric component with solid PZT-5A as the base material and a required volume fraction $V_{\max}=0.5$. We see that the optimisation algorithm performs in a similar manner to previously published work [29]. In particular, the explicit post-processing optimisation problem to remove intermediate densities from the optimised design works effectively. Furthermore, we find that the penalisation exponents of 4, 5 and 5 on the stiffness, piezoelectric and dielectric tensors is suitable [29].

**Figure 8:**
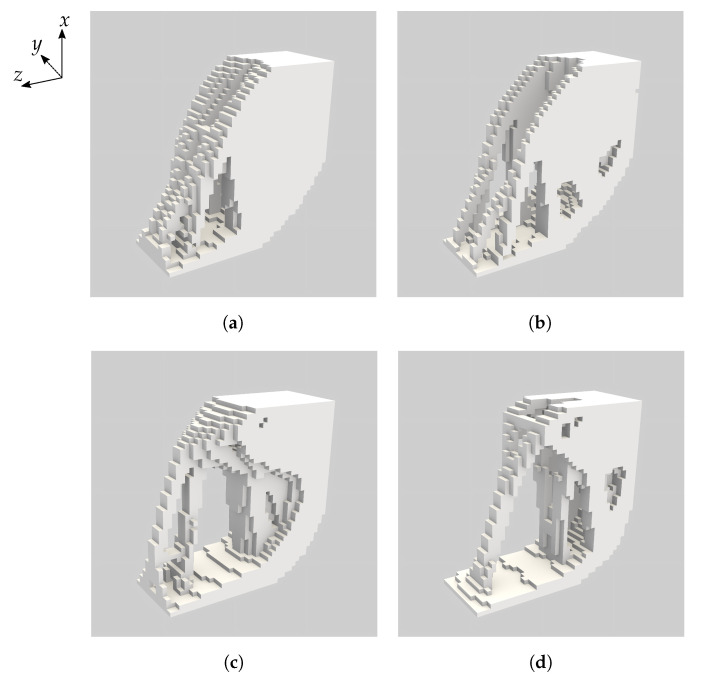
Visualisations of the four topology optimised piezoelectric components. (**a**) Base material PZT-5A with maximum volume fraction 0.5. (**b**) Base material PZT-5A with maximum volume fraction 0.35. (**c**) Open-cell optimised PZT-5A base material with maximum volume fraction 0.5. (**d**) Open-cell optimised PZT-5A base material with maximum volume fraction 0.35. The images visualise the presence or absence of base material and we emphasise that the open-cell optimised microstructure (Figure 4) appears where the base material is visualised in (**c**,**d**). The coordinate directions indicated at top left apply to all sub-figures.

**Figure 9:**
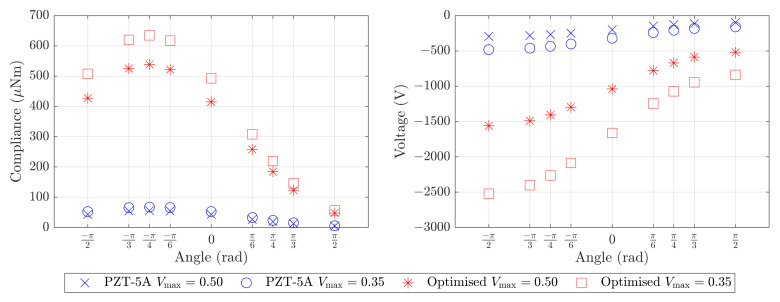
Compliance and voltage computational results for the optimised components presented in Figure 8. The compliance and voltage are shown for a number of angles in addition to the angles of −π/2, 0, and π/2 that were considered in our optimisation problem.

The four topology optimised designs with the two different base materials and two maximum volume fractions are presented in Figure 8. We summarise the mass, compliance and voltage computational results for the optimised designs in Table 2. Figure 9 presents the compliance and voltage results for a range of angles in addition to the angles of −π/2, 0, and π/2 that were explicitly included in the optimisation problem. We give data for these additional angles to assess the suitability of the component for multi-functional applications.

**Figure 10:**
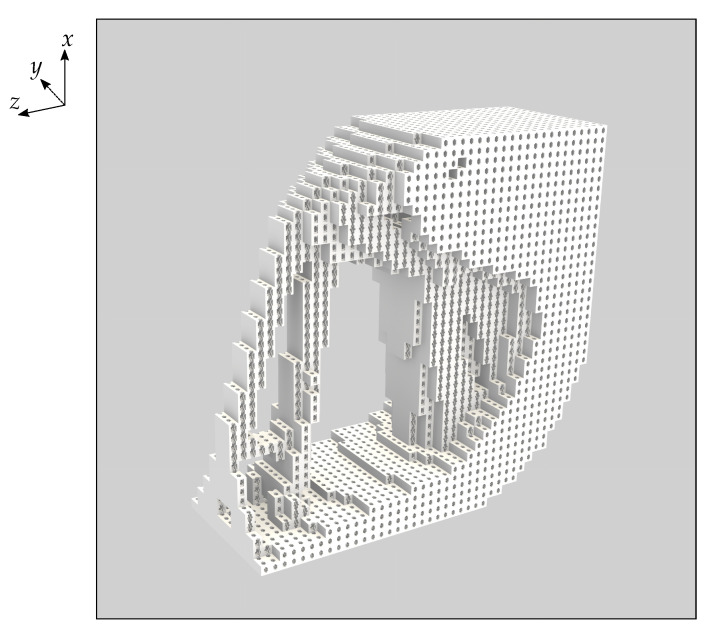
A visualisation of the topology optimised multi-scale piezoelectric component corresponding to Figure 8c and showing the open-cell optimised base material.

It is important to emphasise that in Figure 8c,d the solid phase is constructed from the optimised open-cell base material. To emphasise this we give a visualisation of the $V_{\max}=0.5$ multi-scale topology optimised piezoelectric component from Figure 8c in Figure 10.

### 5.3. Volume Minimised Component

In this section we consider one additional optimisation problem where we minimise the volume of the piezoelectric component while constraining the average compliance and voltage for the three robot configurations. Such a volume minimised design would have low mass and provide a good voltage response. The optimisation problem we consider is:

(32) $$\begin{array}{cc} \underset{\rho_{e}}{\mathrm{minimise}}\;\; & V(\rho_{e})\\ \mathrm{subject}\;\mathrm{to}\; & \sum_{\alpha =1}^{m}a_{\alpha }C_{\alpha }\left(u^{\left(\alpha \right)}\right)\leq C_{\max},\\ \; & \sum_{\alpha =1}^{m}b_{\alpha }J\left(\varphi^{\left(\alpha \right)}\right)\leq \varphi_{\min},\\ \; & h\left(\rho_{e}\right)=l_{\alpha }\left(\rho_{e}\right),\;\forall \alpha =1,\cdots ,m, \end{array}$$

where $C_{\max}$ is the upper bound on the weighted sum of the compliance values, other variables are as described previously, and the base material is choosen to be PZT-5A.

**Figure 11:**
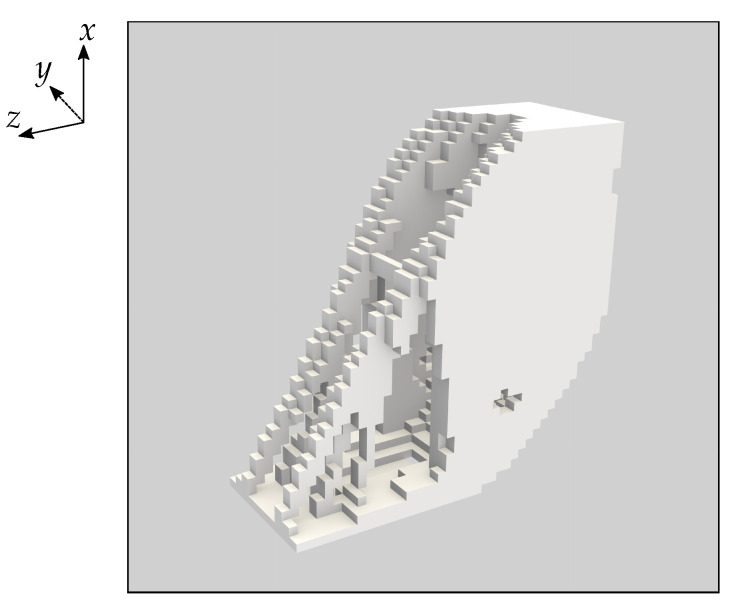
Visualisation of a topology optimised piezoelectric component with minimised volume. The base material is PZT-5A and volume minimisation is subject to constraints on the average compliance and voltage for the three inclination angles of −π/2, 0 and π/2. This optimisation result has volume fraction 0.295 and properties as in Table 3.

We solve this topology optimisation problem with a compliance constraint of $C_{\max}=50$ μNm. This $C_{\max}$ value is slightly smaller than the average compliance calculated for the reference component with PZT-5A as the base material. We choose a slightly lower value for the compliance constraint than that of the reference component because a higher computational resolution is required to effectively explore lower volume fraction designs. In Figure 11 and Table 3 we present the results for this volume minimisation problem. The volume fraction of the optimised design is 0.295.

## 6. Discussion

In this section we discuss the properties of our presented optimised piezoelectric components in comparison to the reference component and reflect on the design objectives described in the Introduction. We summarise the main design objectives as follows:

- Mechanical properties: lightweight and low compliance.
- Sensing capability: piezoelectric sensing capability.
- Robustness to climbing configurations: functional for inclination angles θ of −π/2, 0, and π/2.

Focussing first on the mass of the reference and optimised components, we see that the reference component with ABS as the base material is the lightest of the presented components with a mass of 3.218 g (see Table 1). The open-pore microstructure of the optimised base material results in a smaller mass for the reference and optimised components with this base material compared to solid PZT-5A (see Table 1 and Table 2). The optimised piezoelectric component with solid PZT-5A base material and $V_{\max}=0.50$ has the largest mass of all presented components (23.80 g, see Table 2). While this mass is significantly larger than that of the existing ABS part, we emphasise the purpose of the optimised piezoelectric components is to enable replacement of the off-the-shelf force sensor currently included within each foot of MAGNETO. These force sensors have a mass of 120 g, meaning that removing the force sensor and using an optimised PZT-5A design at 50% volume fraction would achieve a weight saving of around 60% compared to the force sensor when accounting for two optimised components per foot. This significant potential for saving weight was achieved by including a constraint on the volume fraction within the optimisation problem.

The relatively low Young’s modulus of ABS results in a high compliance for the ABS reference component. It is clear from the left plot of Figure 6 that using PZT-5A solid material or PZT-5A open-cell optimised base material significantly reduces the compliance of the reference component compared to ABS. Our optimised designs using PZT-5A and the optimised open-cell material improve upon the compliance of the reference component with the same base material significantly (see Table 1 and Table 2). This reduction in compliance is possible even with the low volume fraction optimised components ($V_{\max}=0.35$): despite the lower volume fraction, optimisation of the compliance objective exploits the available design freedom to improve the compliance compared to the reference component.

Piezoelectric sensing capability of the optimised components was achieved by imposing an inequality constraint on the average voltage $\varphi (x_{0})$ measured for the inclinations −π/2, 0 and π/2. The constraint value was chosen to be the average voltage for the inclinations −π/2, 0 and π/2 for the reference component with PZT-5A as the base material (−137 volts, see Table 1). All of the optimised components improved upon this average voltage significantly (see Table 2). The average voltage improvement was more than 40% in the case of PZT-5A with $V_{\max}=0.50$ and more than 650% in the case of the open-cell optimised PZT-5A material with $V_{\max}=0.50$. The increase in the voltage for both cases is likely due to the thin rods of material in the optimised components that connect the load-bearing boundary $\Gamma_{I}$ to the rest of the structure. This is evident in all four designs presented in Figure 8. The $V_{\max}=0.35$ optimised components perform even better than the $V_{\max}=0.50$ components in terms of voltage sensitivity. This is clear from the values in Table 2 and also exemplified in the right hand plot in Figure 9. These voltage results suggest that the optimised piezoelectric components have the potential to replace the heavy off-the-shelf force sensors in each foot of MAGNETO, however additional research is required to determine the effective sensing range of these components.

The optimised piezoelectric components need to be robust to a range of climbing configurations, and particularly for the inclination angles θ of −π/2, 0, and π/2 that relate to the three actuation cases of horizontal movement, vertical movement, and inverted horizontal movement. Comparing the values in Table 1 and Table 2 show that the optimised piezoelectric components give compliance and voltage results that are better than the reference configuration results for the same base material for all three optimised inclination angles and both maximum volume fractions. Figure 6 and Figure 9 show the compliance and voltage for several additional inclination angles. We see that all components follow a similar pattern of higher compliance and voltage sensitivity for inclination angles in the range −π/2≤θ≤0. This occurs due to the significant cancellation of the two stresses acting on $\Gamma_{l}$ in inverted configurations (0<θ<π/2). Overall, the optimised components have robust behaviour for a variety of loading configurations.

We considered one additional topology optimisation problem where we minimised the volume of the component while constraining the average compliance and voltage to be similar to the PZT-5A reference component. The optimised design presented in Figure 11 and the corresponding values in Table 3 again demonstrate improved voltage and weight compared to the other presented configurations with PZT-5A as the base material. The design has a volume fraction of 0.295, and corresponding mass of 14.38 g. Designs with even lower volume fraction could potentially be achieved with a higher resolution for the design domain and would require additional computational resources. We note that in general it can be difficult to choose an appropriate constraint value for the compliance if there is no reference configuration.

The above discussion demonstrates that our optimised piezoelectric components satisfy all three design criteria. However, there are clear trade-offs that would need to be considered to choose a single best design. For example, using the optimised open-cell base material gives a significant improvement in potential sensing capability and would be lighter in weight compared to a design using solid PZT-5A as the base material. However, an open-cell design would also be more compliant, more difficult to manufacture, and may not have the mechanical strength needed for use in the robotic application. Experimental research including building of prototypes would be needed to evaluate these other aspects of each of the designs. It would also be possible to further enhance the component’s sensing capability by considering the poling direction of piezoelectric base material as a design variable. This idea is closely related to the consideration of material orientation when using anisotropic base materials, e.g., [37]. Material orientation is typically considered using either a discrete [38] or continuous [39] set of orientations. This could be considered in future work.

Finally, while our work has focussed on the computational analysis of possible piezoelectric component designs, we briefly discuss manufacturability. Recent research has demonstrated the successful additive manufacture of piezoelectric materials [14]. Such technology would enable the manufacture of our optimised designs with PZT-5A as the base material. The manufacturing technique proposed by Cui et al. [14] could also be applied to the construction of our designs utilising the open-cell optimised base material. Experimental research would be needed to evaluate the mechanical and piezoelectric properties of such prototypes and their reliability for extended use.

## 7. Conclusions

In this paper we have considered the computational design of a multi-functional, lightweight, integrated piezoelectric force sensor for the foot of a climbing robot. We consider the average compliance and measured voltage of the designed component for three robot configurations. The design problem is formulated in three dimensions, without restriction to two dimensions or a layered structure. Furthermore, we consider both a solid base material and the use of an architectured piezoelectric material as the base material in the optimisation problem. Our optimised designs provide improved compliance and piezoelectric sensing capability compared to a reference design. The designs would reduce robot weight and increase payload capacity if they can replace existing off-the-shelf force sensors. Overall, we have demonstrated the successful computational optimisation of three-dimensional integrated piezoelectric sensing components for a climbing robot. Such components are worthy of future experimental investigation.

## Figures and Tables

**Table 1:** Reference component computational results for ABS, PZT-5A, and the open-cell optimised material made of PZT-5A. The results give the compliance and voltage for each inclination θ of −π/2,0 and π/2, along with the average for all three configurations. We also give the computed mass of the reference component for each base material.

Material	θ	Compliance (μNm)	Voltage (V)
ABS	−π/2	2287	0
3.218 g	0	2047	0
	π/2	254.1	0
	Avg:	1529	0
PZT-5A	−π/2	87.24	−208.2
22.82 g	0	79.25	−133.4
	π/2	9.694	−69.41
	Avg:	58.73	−137.0
Optimised (Open)	−π/2	959.9	−909.5
11.41 g	0	856.7	−597.8
	π/2	106.6	−303.2
	Avg:	641.1	−603.5

**Table 2:** Computational mass, compliance and voltage results for the four topology optimised piezoelectric components presented in Figure 8. The four components have solid PZT-5A and the open-cell optimised PZT-5A as base materials at maximum volume fractions of 0.5 and 0.35.

Case	θ	Compliance (μNm)	Voltage (V)
PZT-5A	−π/2	43.59	−296.1
$V_{\max}=0.5$	0	43.33	−199.8
23.80 g	π/2	4.844	−98.71
	Avg:	30.59	−198.21
Optimised (Open)	−π/2	427.1	−1561
$V_{\max}=0.5$	0	414.4	−1037
11.93 g	π/2	47.45	−520.4
	Avg:	296.3	−1039
PZT-5A	−π/2	52.59	−482.8
$V_{\max}=0.35$	0	52.79	−321.2
16.84 g	π/2	5.843	−160.9
	Avg:	37.07	−321.7
Optimised (Open)	−π/2	510.1	−2544
$V_{\max}=0.35$	0	495.4	−1675
8.372 g	π/2	56.68	−848.1
	Avg:	354.0	−1689

**Table 3:** Computational mass, compliance and voltage results for the volume minimised piezoelectric component presented in Figure 11. The component has solid PZT-5A as the base material and a volume fraction of 0.295.

Mass	θ	Compliance (μNm)	Voltage (V)
14.38 g	−π/2	59.83	−597.0
	0	61.40	−399.7
	π/2	6.647	−199.0
	Avg:	42.62	−398.6

## Data Availability

Important data are contained within the article. Additional data may be available upon reasonable request to the corresponding author.
